# Supplementary material for: HIVprotI: an integrated web based platform for prediction and design of HIV proteins inhibitors
Source: J Cheminform. 2018 Mar 9;10:12. doi: 10.1186/s13321-018-0266-y (PMC5845081; doi:10.1186/s13321-018-0266-y)
Supplement: Supplementary file 2 — Additional file 2. Source code of HIVProtI web server. [file 13321_2018_266_MOESM2_ESM.zip › HIVprotI_Source-code/hivprotI/jsme/jme_examples/jme_example1.html]

JME Example 1


## Example of Java - JavaScript communication and data export / import

You have to enable Java and JavaScritpt on
your machine !

JME
Help

**smiles**  
   

**jme string**  


**mol file**

       

**Keyboard shortcuts**

| bonds -,=,# | rings 3..8, Ph is 1, furyl 0 | atoms C,N,O,P,S,F,L,B,I,H | groups t - tert.butyl, ft - CF3, y - nitro, a - COOH, z - SO3H |
| d - delete | Esc - return to the standard state (C, single bond) | | || molecule may by moved by "dragging" free space and rotated by pressing concurrently also the shift key | | | |
